# Striatal dopamine synthesis and cognitive flexibility differ between hormonal contraceptive users and nonusers

**DOI:** 10.1093/cercor/bhad134

**Published:** 2023-05-09

**Authors:** Caitlin M Taylor, Daniella J Furman, Anne S Berry, Robert L White, William J Jagust, Mark D’Esposito, Emily G Jacobs

**Affiliations:** Department of Psychological & Brain Sciences, University of California, Santa Barbara, CA 93106, United States; Department of Neurology, University of California San Francisco, San Francisco, CA 94143, United States; Department of Psychology, Brandeis University, Waltham, MA 02453, United States; Department of Neurology, Washington University School of Medicine, St. Louis, MO 63112, United States; Helen Wills Neuroscience Institute, University of California Berkeley, Berkeley, CA 94720, United States; Lawrence Berkeley National Laboratory, Berkeley, CA 94720, United States; Helen Wills Neuroscience Institute, University of California Berkeley, Berkeley, CA 94720, United States; Department of Psychology, University of California Berkeley, Berkeley, CA 94720, United States; Department of Psychological & Brain Sciences, University of California, Santa Barbara, CA 93106, United States; Neuroscience Research Institute, University of California Santa Barbara, Santa Barbara, CA 93106, United States

**Keywords:** cognitive flexibility, dopamine, hormonal contraception, PET imaging

## Abstract

In rodents and nonhuman primates, sex hormones are powerful modulators of dopamine (DA) neurotransmission. Yet less is known about hormonal regulation of the DA system in the human brain. Using positron emission tomography (PET), we address this gap by comparing hormonal contraceptive users and nonusers across multiple aspects of DA function: DA synthesis capacity via the PET radioligand 6-[^18^F]fluoro-m-tyrosine ([^18^F]FMT), baseline D2/3 receptor binding potential using [^11^C]raclopride, and DA release using methylphenidate-paired [^11^C]raclopride. Participants consisted of 36 healthy women (*n* = 15 hormonal contraceptive users; *n* = 21 naturally cycling/non users of hormonal contraception), and men (*n* = 20) as a comparison group. A behavioral index of cognitive flexibility was assessed prior to PET imaging. Hormonal contraceptive users exhibited greater DA synthesis capacity than NC participants, particularly in dorsal caudate, and greater cognitive flexibility. Furthermore, across individuals, the magnitude of striatal DA synthesis capacity was associated with cognitive flexibility. No group differences were observed in D2/3 receptor binding or DA release. Analyses by sex alone may obscure underlying differences in DA synthesis tied to women’s hormone status. Hormonal contraception (in the form of pill, shot, implant, ring, or intrauterine device) is used by ~400 million women worldwide, yet few studies have examined whether chronic hormonal manipulations impact basic properties of the DA system. Findings from this study begin to address this critical gap in women’s health.

Sex hormones are powerful neuromodulators of learning and memory (Taxier et al. 2020). Accumulating evidence suggests that sex hormones’ influence extends to the regulation of dopamine (DA) (Becker 1999; Barth et al. 2015; Sun et al. 2016; Yoest et al. 2018), itself a neuromodulator of higher order cognitive functions (Iversen and Iversen 2007; Cools et al. 2008; Cools and Arnsten 2022). In rodents and nonhuman primates, 17β-estradiol (E2) and progesterone (P) modulate DA synthesis and release, alter DA-D2 receptor availability, and modify the basal firing rate of dopaminergic neurons (Dluzen and Ramirez 1984, 1990; Becker and Cha 1989; Lévesque et al. 1989; Pasqualini et al. 1995; Czoty et al. 2009; Asghari et al. 2011). For example, E2 administration produces a dose-dependent increase in striatal DA (Pasqualini et al. 1995) and modulates goal-directed behavior (Uban et al. 2012) in rodents. Progesterone has a bimodal effect on striatal DA concentration, with increases in DA in the first 12 h after P perfusion, and inhibitory effects 24 h post-infusion. Furthermore, surgical removal of the ovaries reduces tyrosine hydroxylase (TH)-immunoreactive neurons in the substantia nigra (Leranth et al. 2000) and prefrontal cortex (PFC; Kritzer and Kohama 1998). Estrogen receptors are localized to regions that receive major projections from midbrain DA neurons, including PFC, dorsal striatum, and the nucleus accumbens (Björklund and Dunnett 2007). Despite the substantial literature supporting sex hormones’ role in DA neuromodulation in rodents and nonhuman primates, less is known about hormonal regulation of the DA system in the human brain.

Indirect evidence in humans suggests that estradiol modulates DA-dependent cognitive function and PFC activity (Jacobs and D’Esposito 2011; Jacobs et al. 2016, 2017; Diekhof et al. 2021). For example, Jacobs and D’Esposito (2011) found evidence that estradiol regulates PFC activity and working memory performance, and the direction of the effect depends on an individual’s basal PFC DA tone (indexed by catechol-O-methyltransferase activity). Additional evidence suggests that menstrual cycle phase influences DA-dependent motor and cognitive functions, including response time on tests of manual coordination, working memory, and cognitive flexibility (Hampson and Kimura 1988; Hidalgo and Pletzer 2017), and immediate reward selection bias (Smith et al. 2014).

Molecular positron emission tomography (PET) imaging provides a more direct assessment of dopaminergic activity in vivo in the human brain. Sex differences have been observed in DA synthesis capacity (Laakso et al. 2002), DA release (Munro et al. 2006; Riccardi et al. 2006; Manza et al. 2022), and DA transporter density (Lavalaye et al. 2000; Mozley et al. 2001). PET studies of women sampled in different phases of the menstrual cycle and menopausal transition suggest a role for sex steroid hormones in modulating aspects of DA functioning. Wong et al. (1988) observed fluctuations in DA-D2 receptor density across the menstrual cycle in healthy premenopausal women, and Pohjalainen et al. (1998) observed a greater variability in DA-D2 receptor density in premenopausal vs postmenopausal women, with the suggestion that greater variability was attributable to hormonal fluctuations across the menstrual cycle (but see Nordström et al. 1998; Smith et al. 2019; Petersen et al. 2021).

An underexplored population for studying hormonal influences on DA function is women using hormonal contraception. Hormonal contraception (HC; in the form of pill, shot, implant, ring, or intrauterine device (IUD)) is used by ~400 million women worldwide (United Nations 2019), yet few studies have examined whether chronic hormone manipulations affect basic properties of the DA system. In the present analyses, we leveraged data from a well-characterized cohort (Berry et al. 2018a, 2019; Furman et al. 2020, 2021) to probe the impact of hormonal contraception on multiple properties of the DA system using molecular PET imaging techniques, offering new insights into the relationship between endocrine status and DA neurotransmission in the human brain. The study consisted of young, healthy women and men, and paired pharmacological manipulation of the DA system with PET imaging to assess synthesis capacity (radioligand [^18^F]fluoro-m-tyrosine, [^18^F]FMT), D2 receptor availability (radioligand [^11^C]raclopride), and DA release (radioligand [^11^C]raclopride paired with methylphenidate). This provides a unique opportunity to characterize differences in DA synthesis capacity, basal DA receptor occupancy, and stimulated DA release in a single cohort. Next, we investigated sex differences in DA neurotransmission. Finally, we examined whether differences in DA neurotransmission were associated with DA-dependent cognition, using a behavioral assessment of cognitive flexibility (Berry et al. 2018b; Furman et al. 2020). Leveraging an existing multimodal molecular PET imaging cohort allowed us to look at broad differences in DA neurotransmission between naturally cycling women (i.e. those who self-report not using any hormone-based medication) and those using hormonal contraception. These results offer researchers a strong rationale for designing additional imaging studies that explicitly test the breadth and depth with which hormonal contraceptives influence major neuromodulatory systems.

## Materials and methods

### Participants

Participants consisted of 57 adults (mean age = 21.16 years, SD = 2.37, range: 18–28 years), including 37 women and 20 men (*n* = 28 Asian, 10 Hispanic or Latino, 9 White (not Hispanic or Latino), 2 Black or African American, 3 more than one race or ethnicity, 2 other, and 3 declined to state). Participants were originally recruited and underwent PET and MRI imaging as part of a parent study on dopaminergic mechanisms of cognitive control (e.g. see Furman et al. 2020). PET data from this sample have previously been described in Berry et al. (2018a). This study was approved by Institutional Review Boards at the University of California, Berkeley and Lawrence Berkeley National Laboratory, and the experiments were undertaken with the understanding and written consent of each subject. Data captured regarding participants’ hormonal contraceptive use allowed for post hoc comparisons between current users and current non-users of hormonal contraception. Participants met the following eligibility criteria: (i) 18–30 years old, (ii) right-handed, (iii) current weight of at least 100 pounds, (iv) able to read and speak English fluently, (v) nondrinker or light drinker (women: <7 alcoholic drinks/week; men: <8 alcoholic drinks/week), (vi) no recent history of substance abuse, (vii) no history of neurological or psychiatric disorder as confirmed by clinician interview, (viii) no current psychoactive medication or street drug use, (ix) not pregnant, and (x) no contraindications to MRI. Most participants completed 3 PET scans over the course of 2 separate sessions: [^18^F]FMT, and [^11^C]raclopride + placebo and [^11^C]raclopride + methylphenidate on the same day; the exceptions were 1 participant (an NC woman) who did not complete the FMT scan due to technical issues, 1 participant (an NC woman) who did not produce reliable Raclopride scan data due to technical issues, and 2 participants (hormonal contraceptive users) who did not complete Raclopride scans.

Participants also completed a listening span test (Salthouse and Babcock 1991) and the Barratt Impulsivity Scale (Patton et al. 1995) to assess working memory capacity and trait impulsivity, respectively.

#### [^18^F]FMT sample

Women were categorized based on hormone status: naturally cycling (NC, no current reported use of hormonal contraception; *n* = 21, avg. age = 22.67 years, SD = 2.77) and current users of hormonal contraception (HC, *n* = 15, avg. age = 20.43 years, SD = 1.91). Types of hormonal contraception used included: combined oral contraception (OC, *n* = 10), vaginal ring (*n* = 1), implant (*n* = 2), injection (*n* = 1), and hormonal IUD (*n* = 1).

#### [^11^C]Raclopride sample

[^11^C]Raclopride data from 1 NC participant did not pass quality control and 2 HC users (combined OC) did not have [^11^C]raclopride sessions, yielding a final sample of 21 NC women (avg age = 20.67, SD = 1.91) and 13 HC users (avg age = 22.69, SD = 2.81).

In our secondary analyses, participants were grouped by self-reported sex (male, *n* = 20; female, *n* = 37), and hormone status (male, NC, and HC). Men and women did not differ significantly in age or BMI; however, HC users were older than males (*p* = 0.03, *d* = 0.85) and NC participants (*p* = 0.01, *d* = 0.94) by 25 months on average (Table 1).

**Table 1:** Participant demographics by sex and hormone status.

	Age	BMI
**Men** (*n* = 20)	20.7 ± 2.1	23.8 ± 5.3
**Women** (*n* = 37)	21.4 ± 2.5	23.7 ± 4.2
***Naturally Cycling*** (*n* = 22)	20.6 ± 2.0	23.0 ± 3.9
***Hormonal *** ***Contraception*** (*n* = 15)	22.7 ± 2.8	24.7 ± 4.6
**NC vs HC** Cohen’s *d* (Welch’s *p*)	0.94 (0.01^a^)	n.s.
**Men vs Women**	n.s.	n.s.
**Men vs NC vs HC** Kruskal–Wallis *p*	0.03	n.s.

Types of HC used (*n*): combined OC (10), vaginal ring (1), implant (2), injection (1), and hormonal IUD (1). NC, naturally cycling; HC, hormonal contraception.

^a^Indicates significance with Bonferroni correction (*P* < 0.0167).

As trait-like measures of working memory span, impulsivity, and smoking status have been associated with striatal DA neurotransmission (Landau et al. 2009; Smith et al. 2018; Ashok et al. 2019), we also considered whether our groups differed by these phenotypes. Neither baseline working memory (listening span score) *F*(2) = 0.27, *p* = 0.77 (HC: mean = 3.07, SD = 0.98; NC: mean = 2.91, SD = 0.81; Male: mean = 2.85, SD = 0.89), nor trait impulsivity (Barrett Impulsivity Scale score) *F*(2) = 0.50, *p* = 0.61 (HC: mean = 55.80, SD = 8.75; NC: mean = 57.86, SD = 5.86; Male: mean = 58.60, SD = 10.25) differed between groups; and a previous characterization of participants from this parent data set found a very low incidence of nicotine use (Berry et al. 2018a).

### Structural MRI scan

Images were acquired using a Siemens 3T Trio Tim scanner with a 12-channel coil. Each participant was scanned on 3 occasions using a high-resolution T1-weighted magnetization prepared rapid gradient echo (MPRAGE) whole-brain scan (TR = 2,300 ms; TE = 2.98 ms; FA = 9°; matrix = 240 × 256; FOV = 256; sagittal plane; voxel size = 1 × 1 × 1 mm; 160 slices). The 3 MPRAGE scans were aligned, averaged, and segmented using FreeSurfer version 5.1 (http://surfer.nmr.mgh.harvard.edu/) and the averaged template was used for coregistration with the PET data.

### [^18^F]FMT PET data acquisition

Participants underwent an [^18^F]FMT PET scan to measure DA synthesis capacity. Detailed methods are provided in Berry et al. (2018b). PET data were acquired using a Siemens Biograph Truepoint 6 PET/CT scanner (Siemens Medical Systems, Erlangen, Germany) ~1 h after participants ingested 2.5 mg/kg of carbidopa to minimize the peripheral decarboxylation of [^18^F]FMT. After a short CT scan, participants were injected with ~2.5 mCi of [^18^F]FMT as a bolus in an antecubital vein (M ± SD; specific activity = 947.30 ± 140.26 mCi/mmol; dose = 2.43 ± 0.06 mCi). Dynamic acquisition frames were obtained over 90 min in 3D mode (25 frames total: 5 × 1, 3 × 2, 3 × 3, 14 × 5 min). Data were reconstructed using an ordered subset expectation maximization algorithm with weighted attenuation, corrected for scatter, and smoothed with a 4 mm full width at half maximum kernel.

### [^11^C]Raclopride PET data acquisition

Participants received two [^11^C]raclopride PET scans an average of 21.65 days before or after the [^18^F]FMT scan (median = 7 days) to measure D2/3 receptor occupancy and DA release. To measure baseline D2/3 receptor occupancy, participants ingested a placebo pill ~1 h before [^11^C]raclopride scan 1. The placebo scan was always performed first. To measure DA release, participants ingested 30 mg (M ± SD mg/kg: 0.46 ± 0.08) of methylphenidate ~1 h before [^11^C]raclopride scan 2. Endogenous DA release was measured as the percent signal change (PSC) in nondisplaceable binding potential (BPND) from [^11^C]raclopride scans 1 to 2 ((placebo [^11^C]raclopride − methylphenidate [^11^C]raclopride)/placebo [^11^C]raclopride). Scans were conducted on the same day, 2 h apart and participants were blind to whether placebo or methylphenidate was administered. For both [^11^C]raclopride scans 1 and 2, after a short CT scan, participants were injected with ~10 mCi of [^11^C]raclopride as a bolus in an antecubital vein. Dynamic acquisition frames were obtained over 60 min in 3D mode (19 frames total: 5 × 1, 3 × 2, 3 × 3, 8 × 5). Reconstruction was performed as described above.

### PET data analysis

PET data were preprocessed using SPM8 software (Friston et al. 2007). To correct for motion between frames, images were realigned to the middle frame. The first 5 images were summed prior to realignment to improve realignment accuracy, as these early images have relatively low signal contrast. Structural images were coregistered to PET images using the mean image of frames corresponding to the first 20 min of acquisition as a target. The mean image for the first 20 min was used rather than the mean image for the whole scan time because it provides a greater range in image contrast outside of striatum thus making it a better target for coregistration.

#### [^18^F]FMT

Graphical analysis for irreversible tracer binding was performed using Patlak plotting (Patlak and Blasberg 1985; Sossi et al. 2003) implemented using in-house software and MATLAB version 8.2 (The MathWorks, Natick, MA). Without measurement of the arterial input function, [^18^F]FMT PET analysis used reference region models. Cerebellar gray matter was used as the reference region because this region shows very little tracer uptake, and has an extremely low density of DA receptors and metabolites relative to striatum (Farde et al. 1986; Camps et al. 1989; Levey et al. 1993; Hall et al. 1994). The most anterior, one-fourth of cerebellar gray matter, was removed from the reference region to limit contamination of signal from the substantia nigra and ventral tegmental area. K_i_ images were generated from PET frames corresponding to 25–90 min (Ito et al. 2006, 2007), which represent the amount of tracer accumulated in the brain relative to the reference region.

#### [^11^C]Raclopride

For [^11^C]raclopride PET, reversible tracer binding was quantified using simplified reference tissue model analysis (SRTM; Lammertsma and Hume 1996). Specifically, a basis function version of the SRTM was applied as previously described (Gunn et al. 1997) with posterior cerebellar gray matter used as the reference region. The SRTM analysis was performed using in-house software provided by Dr. Roger Gunn and MATLAB version 8.2. SRTM analysis was used to determine BP_ND_, which can be defined as: BP_ND_ = f_ND_B_avail_/K_D_ where B_avail_ is the concentration of D2/3 receptors, K_D_ is the inverse of the affinity of the radiotracer for D2/3 receptors, and f_ND_ is the free fraction of the ligand in the nondisplaceable tissue compartment (Slifstein and Laruelle 2001; Innis et al. 2007).

### Regions of interest

An ROI approach was used to test relationships between hormonal status and PET measures of dopaminergic function in striatal subregions. Striatal subregions were manually drawn in native space on each participant’s averaged MPRAGE MRI scan using Mango software. The dorsal caudate, dorsal putamen, and ventral striatum were segmented as described in Mawlawi et al. (2001). Inter-rater reliability was high for manually drawn striatal subregions (see Berry et al. 2018b).

As we did not hypothesize an effect of hemisphere, ROI values for our 3 ROIs (dorsal caudate, dorsal putamen, and ventral striatum) were analyzed as voxel-weighted averages of left and right hemisphere PET values as follows:

(*L* value × *L* ROI volume + *R* value × *R* ROI volume)/Combined *L* + *R* ROI volume.

All analyses on striatal values were conducted on partial volume corrected ROIs (PVC; Rousset et al. 1998). PVC was performed in native space (non-normalized data) and corrects for between-subject differences in the inclusion of white matter and CSF in the measured volumes. To apply the PVC in native space, we used FreeSurfer-generated ROIs for gray matter cortical and subcortical regions, white matter, and cerebral spinal fluid. All statistical analyses were conducted using R (version 1.2.5001).

### Cognitive paradigm

The task was an adaptation of the task-switching paradigm developed by Armbruster et al. (2012) and is described in detail in Furman et al. (2020). Briefly, on each trial, participants were required to respond quickly to digits between 1 and 9 (excluding 5) that appeared in different shades of gray against a black background. On 82% of trials, a single digit appeared above a central fixation. For these “ongoing task” trials, participants performed an operation (odd/even or low/high decisions) on the digit and responded by pressing the index finger of either their left or right hand. On the remaining 18% of trials, 2 digits appeared on the screen simultaneously: 1 above and 1 below the fixation cross. The relative brightness of the upper and lower digits varied and encoded a task cue. When the upper digit was brighter (6% of trials), participants were instructed to ignore the lower digit and continue to apply the ongoing task rule to the upper digit (“distractor trials”). When the lower digit was brighter (6% of trials), participants were signaled to switch attention to the lower digit and to apply the alternate task rule to it (“switch trials”). On the final third of these trials (6% of total trials), the difference in brightness between the upper and lower digits was reduced (“ambiguous trials”). Ambiguous trials were not considered in our analyses. Participants performed a total of 990 trials distributed across 3 blocks with brief interposed breaks. Cognitive testing occurred prior to PET imaging. *Distractor cost* was calculated as the difference between performance accuracy on “distractor” trials and “ongoing” trials, and is thought to reflect cognitive stability, a process sensitive to variation in prefrontal DA. *Switch cost* was calculated as the difference between performance accuracy on “switch” vs “ongoing” trials and is a putative behavioral marker of cognitive flexibility, reflective of striatal DA. One NC participant did not undergo cognitive testing, resulting in a final sample of 20 NC women (avg age = 20.67, SD = 1.91), 15 HC users (avg age = 22.69, SD = 2.81), and 20 men (avg age = 20.70, SD = 2.08).

### Statistical analysis

#### Impact of hormone status on DA neurotransmission

Since hormonal contraceptive (HC) users were older than NC participants, to compare markers of dopaminergic signaling between HC and NC groups, we conducted 2 × 4 ANCOVA (hormone group × bilateral region of interest, controlling for the effects of age) for measures of interest (FMT K_i_, [^11^C]raclopride BP_ND_ and PSC in [^11^C]raclopride BP). We investigated significant main effects with post hoc 1-way ANCOVAs to determine which regions were driving the effect, controlling for the effects of age. Statistically significant findings that survived Bonferroni correction for multiple comparisons are noted (*p_Bf_* = 0.05/3 regions = 0.0167). Partial effect sizes ($\eta$^2^) are reported for statistically significant findings.

Welch’s *t*-tests were used to compare distractor costs and switch costs between our comparison groups. One NC participant with unusable task data was omitted from these analyses. As a follow-up to observed differences between NC and HC women, switch costs were correlated with [^18^F]FMT K_i_ PVC striatal values to evaluate a relationship between performance and DA synthesis.

#### Sex differences in DA neurotransmission

To compare the aspects of DA signaling by sex and hormone status, we conducted 2 × 3 mixed ANCOVA (group × bilateral region of interest, controlling for age) for measures of interest (FMT K_i_, [^11^C]raclopride BP, and PSC in [^11^C]raclopride BP).

Welch’s *t*-tests were conducted to compare distractor costs and switch costs by sex. Additionally, for reference, switch costs were correlated with [^18^F]FMT K_i_ PVC striatal values in our male sample.

Finally, to determine whether differences in hormonal status within women influenced the detection of sex differences, we conducted 3 × 3 mixed ANCOVA (group × bilateral region of interest, controlling for age) for each measure of interest (FMT K_i_, [^11^C]raclopride BP, and PSC in [^11^C]raclopride BP). Significant main effects were investigated using post hoc 1-way ANCOVAs, again to control for the effects of age.

## Results

### DA neurotransmission differs with hormonal contraceptive use

#### Striatal [^18^F]FMT K_i_

**Fig. 1:**
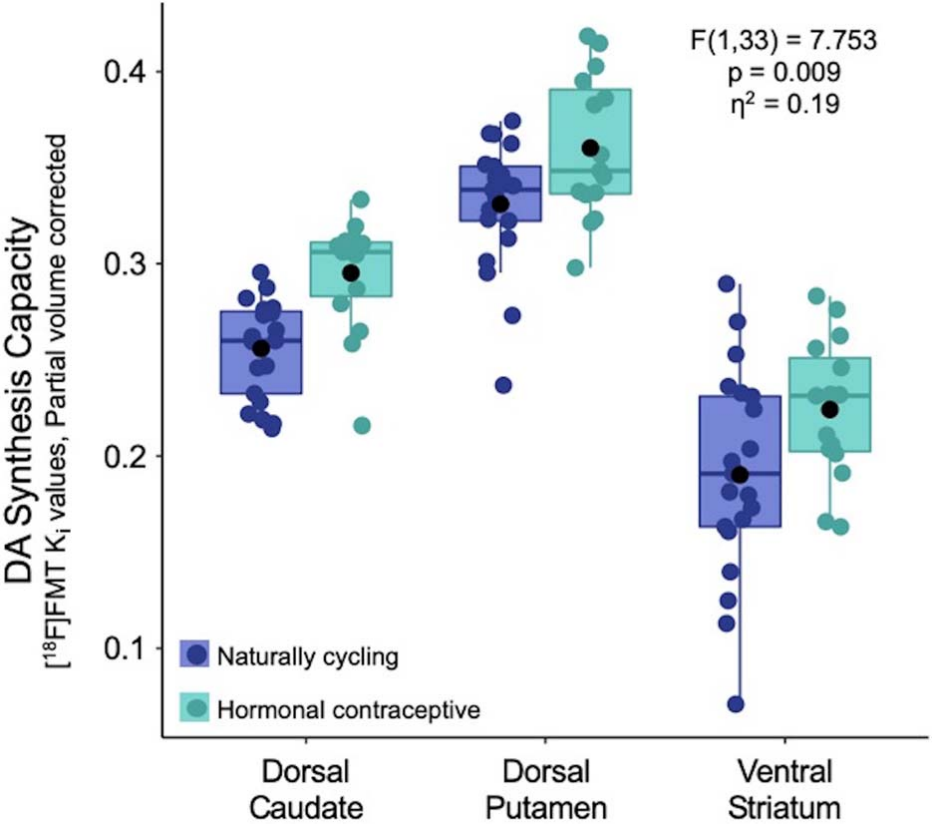
Effect of hormone status on DA synthesis capacity. [^18^F]FMT K_i_ values in naturally cycling females and hormonal contraceptive users by striatal region of interest. Striatal DA synthesis capacity was greater in hormonal contraceptive users relative to naturally cycling women, with the most pronounced effects observed in dorsal caudate.

[^18^F]FMT PET data were obtained to assess DA synthesis capacity in the striatum. ANCOVA revealed significant main effects of age (*F*(1,33) = 4.844, *p* = 0.035, $\eta$^2^ = 0.13), hormone status (*F*((1,33) = 7.753, *p* = 0.009, $\eta$^2^ = 0.19, Fig. 1), and region (*F*(2,68) = 207.859, *p* < 0.00001, $\eta$^2^ = 0.86). Regional effects were expected as previously reported (Berry et al. 2018a). There was no significant interaction between hormone status and region. Results from post hoc 1-way ANCOVAs indicate that hormonal contraceptive users exhibited greater FMT K_i_ values compared with NC participants, with the largest effect in dorsal caudate (*F*(1, 33) = 9.611, *p_Bf_* = 0.004). K_i_ values differed marginally between hormonal contraceptive users and NC participants in dorsal putamen (*F*(1,33) = 3.966, *p* = 0.055) and ventral striatum (*F*(1,33) = 3.754, *p* = 0.061) (Table 2; see Supplementary Table 2 for uncorrected values).

**Table 2:** Dopamine synthesis capacity ([^18^F]FMT K_i_ values) by group and striatal region of interest.

	**Dorsal caudate**	**Dorsal putamen**	**Ventral striatum**
**Male**	0.0278 ± 0.0034	0.0346 ± 0.0030	0.0209 ± 0.0034
**Women**	0.0272 ± 0.0033	0.0343 ± 0.0037	0.0204 ± 0.0049
***Naturally Cycling***	0.0256 ± 0.0025	0.0331 ± 0.0033	0.0190 ± 0.0053
***Hormonal Contraception***	0.0295 ± 0.0030	0.0360 ± 0.0037	0.0224 ± 0.0037
**HC vs NC** partial $\eta$^2^, *p*-value	0.23, 0.004^a^	0.11, 0.055	0.10, 0.061
**Men vs Women** partial $\eta$^2^, *p*-value	0.02, n.s.	<0.01, n.s.	<0.01, n.s.
**Men vs NC vs HC** partial $\eta$^2^, *p*-value	0.18, 0.006^a^	0.08, n.s.	0.10, 0.064

^a^Indicates significance with Bonferroni correction (*p* < 0.0167). n.s. indicates *p* > 0.10. NC, naturally cycling; HC, hormonal contraception.

#### Striatal [^11^C]raclopride BP

[^11^C]Raclopride PET data were obtained to measure D2/3 receptor binding potential. [^11^C]Raclopride BP differed significantly by region (*F*(2,64) = 389.281, *p* < 0.0001, $\eta$^2^ = 0.92). Regional effects were expected as previously reported (Berry et al. 2018a). There was no significant main effect of age (*F*(1,31) = 3.795, *p* = 0.061) or hormone status (*F*(1,31) = 0.09, *p* = 0.76) on [^11^C]raclopride BP_ND_ values, nor was there an interaction between hormone status and region (*F*(2,64) = 0.815, *p* = 0.447) (see Supplementary Table 1 for values).

#### PSC in striatal [^11^C]raclopride BP

Methylphenidate-paired [^11^C]raclopride PET data were acquired to measure DA release. [^11^C]Raclopride BP PSC values differed significantly by region (*F*(2,64) = 389.281, *p* < 0.0001, $\eta$^2^ = 0.92). Again, regional effects were expected as previously reported (Berry et al. 2018a). There were no significant effects of age (*F*(1,31) = 3.795, *p* = 0.061) or hormone status (*F*(1,31) = 0.092, *p* = 0.76) on [^11^C]raclopride BP PSC values, nor was there an interaction between status and region (*F*(2,64) = 0.815, *p* = 0.45) (see Supplementary Table 1 for values).

### DA neurotransmission does not differ by sex

#### Striatal [^18^F]FMT K_i_

We observed a main effect of region on FMT values (*F*(2,108) = 358.424, *p* < 0.0001, $\eta$^2^ = 0.87), no main effect of sex (*F*(1,53) = 0.415, *p* = 0.52), and no interaction between sex and region (*F*(2,108) = 0.032, *p* = 0.97).

#### Striatal [^11^C]raclopride BP

We observed a main effect of region on [^11^C]raclopride BP values (*F*(2,104) = 479.362, *p* < 0.0001, $\eta$^2^ = 0.90), but no main effect of sex (*F*(1,52) = 0.084, *p* = 0.77), and no interaction between sex and region (*F*(2,104) = 1.453, *p* = 0.24).

#### Striatal [^11^C]raclopride BP PSC

Again, we observed a main effect of region on PSC in [^11^C]raclopride BP values (*F*(2,104) = 5.383, *p* = 0.006, $\eta$^2^ = 0.09), but no main effect of sex (*F*(1,51) = 0.089, *p* = 0.77), and no interaction between sex and region (*F*(2, 104) = 1.488, *p* = 0.23).

### Differences in DA neurotransmission by sex and hormone status

#### Striatal [^18^F]FMT K_i_

Despite notable differences in striatal DA synthesis capacity within women based on hormone status, men did not differ appreciably from women in either hormone group. ANCOVA revealed an overall main effect of group (*F*(2,52) = 5.058, *p* = 0.010, $\eta$^2^ = 0.16) and region (*F*(2,106) = 116.5, *p* < 0.00001, $\eta$^2^ = 0.60). Post hoc Tukey’s HSD test confirmed that the main effect of hormone status was driven by significant differences between NC and hormonal contraceptive groups (*p* = 0.004), with no differences between males vs HC (*p* = 0.20) or vs NC (*p* = 0.12).

#### Striatal [^11^C]raclopride BP

We identified a significant effect of region (*F*(2,102) = 476.183, *p* < 0.0001, $\eta$^2^ = 0.90); however, there was no significant main effect of age (*F*(1,50) = 1.330, *p* = 0.25) or hormone status (*F*(2,50) = 0.044, *p* = 0.96), nor an interaction between the 2 factors (*F*(4,102) = 1.049, *p* = 0.39).

#### Striatal [^11^C]raclopride BP PSC

There was a significant effect of region (*F*(2,102) = 5.284, *p* = 0.007, $\eta$^2^ = 0.09), no significant effect of age (*F*(1,50) = 0.400, *p* = 0.53) or hormone status (*F*(2,50) = 0.081, *p* = 0.92), and no significant interaction between the two (*F*(4, 102) = 0.750, *p* = 0.56).

### Individual differences in DA transmission are tied to differences in cognitive flexibility

#### Naturally cycling vs hormonal contraceptive users

**Fig. 2:**
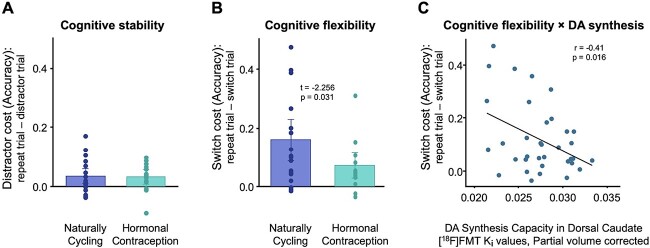
Cognitive flexibility differs between naturally cycling and hormonal contraceptive groups and correlates with DA synthesis capacity in dorsal caudate. A) Performance on a task-switching paradigm reveals no difference in cognitive stability between groups, indicated by no difference in distractor costs on distractor/ongoing trials. B) In contrast, hormonal contraceptive users exhibited greater cognitive flexibility compared with naturally cycling participants, indicated by a smaller performance cost on task-switching trials. C) We observed a significant negative correlation between performance on a task-switching paradigm and [^18^F]FMT Ki values in dorsal caudate across our female participants (both NC and HC).

There was no statistically significant difference in *distractor cost* between hormonal contraceptive users and NC participants (*t*(31.9) = 0.093, *p* = 0.926; Fig. 2a). However, hormonal contraceptive users exhibited significantly reduced *switch cost* compared with NC participants (*t*(31.0) = −2.256, *p* = 0.031; *d* = −0.74; age-adjusted) (Fig. 2b). Across female participants, switch cost was inversely correlated with [^18^F]FMT K_i_ values in the dorsal caudate (Pearson’s *r*(33) = −0.41, *p* = 0.016) and ventral striatum (*r*(33) = −0.34, *p* = 0.042), but not in the dorsal putamen (*r*(33) = −0.29, *p* = 0.089). Only the effect in the dorsal caudate was statistically significant after correcting for multiple comparisons (Fig. 2c). In contrast, there were no significant correlations between [^18^F]FMT K_i_ values and distractor cost in any ROI (all *p*s > 0.6). There were no significant correlations among males between [^18^F]FMT K_i_ values and switch or distractor costs in any ROI (*p* > 0.2 for all).

#### Men vs women

We did not observe a difference in switch cost (*t*(46.4) = −0.11, *p* = 0.91) or distractor cost (*t*(47.9) = −0.47, *p* = 0.64) between men and women (Table 3).

**Table 3:** Performance on task-switching paradigm.

	**Distractor cost**	**Switch cost**
**Men** (*n* = 20)	0.035 ± 0.040	0.125 ± 0.108
**Women** (*n* = 36)	0.029 ± 0.051	0.121 ± 0.132
*** N* *aturally Cycling*** (*n* = 21)	0.029 ± 0.054	0.160 ± 0.149
*** H* *ormonal Contraception*** (*n* = 15)	0.030 ± 0.048	0.070 ± 0.085
**NC vs HC**Cohen’s *d* (Welch’s *p*)	n.s.	−0.74 (0.03)
**Men vs Women**	n.s.	n.s.
**Men vs NC vs HC**Kruskal–Wallis *p*	n.s.	n.s.

NC, naturally cycling; HC, hormonal contraception.

#### Men vs naturally cycling vs hormonal contraceptive users

We did not observe significant effects of switch cost (*F*(2,52) = 2.428, *p* = 0.098) or distractor cost (*F*(2,52) = 0.1, *p* = 0.905) between groups (Table 3).

## Discussion

In this study, hormonal contraceptive users exhibited greater DA synthesis capacity (as measured by [^18^F]FMT K_i_) and greater cognitive flexibility than NC participants. No group differences in D2/3 binding potential ([^11^C]raclopride BP) or DA release ([^11^C]raclopride BP PSC) were observed. Though synthesis capacity differed significantly between NC women and women using hormonal contraception, women overall did not differ appreciably from men. This suggests that investigations into the influence of sex hormones on DA neurotransmission may be hampered if limited to comparisons between sexes. Together, these findings lay the groundwork for understanding how global manipulations of the endocrine system, e.g. via hormonal contraceptives, impact DA neurotransmission and related cognition.

### DA synthesis capacity differs by hormone status

No sex differences in DA neurotransmission were observed. These findings are inconsistent with previous studies comparing DA synthesis between sexes, which report greater synthesis in females relative to males (Laakso et al. 2002; Hahn et al. 2021). However, these studies did not control for or exclude participants based on hormonal contraceptive use. In the present study (e.g. Supplementary Fig. 1), DA synthesis values for our male sample fell between values observed for HC and NC women. Thus, based on findings reported here, the magnitude and direction of a sex effect observed in DA synthesis may reflect the proportion of HC vs NC female participants in the sample rather than a sex difference, per se.

Within women, DA synthesis capacity was greater in hormonal contraceptive users compared with NC participants, whereas D2/3 receptor binding potential and stimulated DA release did not differ between groups. Findings from the preclinical literature suggest that pharmacological manipulations of the hypothalamic-pituitary-gonadal axis impact the DA system. In an ablation-replacement study in ovariectomized rats (Pasqualini et al. 1995), 17β-estradiol add-back selectively increased striatal DA synthesis but not release, as measured via local superfusion of E2 into the caudate nucleus. Similarly, Algeri et al. (1976) observed increased DA synthesis in the striatum and forebrain of intact rats after acute (4 days) and chronic (30 days) oral administration of a synthetic progestin and an estrogen.

Estradiol’s influence on DA synthesis capacity may be mediated by estradiol-induced increases in phosphorylation of tyrosine hydroxylase (TH) (Pasqualini et al. 1995), the enzyme that converts tyrosine to L-dihydroxyphenylalanine (L-DOPA). Another mechanism of action may be the hormonal regulation of aromatic L-amino acid decarboxylase (AADC) that converts L-DOPA to DA (and is the target of [^18^F]FMT). AADC activity is dependent on pyridoxal phosphate (PLP), or Vitamin B_6_ (Rahman et al. 1982; Hartvig et al. 1995), a nutrient and coenzyme with intermediate concentrations in basal ganglia (Ebadi 1985) that is reduced, in some cases to the point of deficiency, in HC users (Luhby et al. 1971; Bennink and Schreurs 1974; Wilson et al. 2011; Rios-Avila et al. 2015). If low levels of PLP are associated with reduced AADC activity (Allen et al. 2010), we would expect HC users to exhibit *reduced* [^18^F]FMT binding relative to NC women. We observed the opposite pattern. Without information regarding vitamin B_6_ status for participants, the relationship between PLP and [^18^F]FMT binding remains untested.

The selectivity of our findings to differences in AADC activity (as measured with [^18^F]FMT) and not DA release or D2/3 receptor binding (both measured with [^11^C]raclopride) also suggests the possibility that other catecholamine systems may be impacted. AADC is a critical enzyme in the formation of catecholamines in general, including serotonin (Ebadi 1985). In rodent studies, chronic treatment with oral hormonal contraceptives increases brain levels of tryptophan and serotonin (Baker et al. 1977; Daabees et al. 1981; reviewed in Porcu et al. 2019). Future investigations should clarify whether global sex steroid hormone manipulations alter DA synthesis capacity specifically, or the catecholamine system generally.

While [^18^F]FMT is a straightforward measure of AADC enzyme activity, which should directly reflect DA synthesis, [^11^C]raclopride is a more complex signal. [^11^C]Raclopride combines several measures, including the binding potential or number of D2/3 receptors (B_avail_), and the dissociation constant or how probable the ligand–receptor complex is to dissociate (K_D_). One limitation of our study is that NC participants were not staged according to menstrual cycle phase. DA release and DA-D2 receptor availability vary across the estrus (Becker and Cha 1989; Lévesque et al. 1989; Yoest et al. 2018) and menstrual cycles (Czoty et al. 2009, though see Munro et al. 2006; Petersen et al. 2021). In ovariectomized rodents, 17β-estradiol administration augments striatal D2 receptor density (B_avail_), but does not influence binding affinity (1/K_D_) (reviewed in Di Paolo 1994). Thus, it is possible that differences in DA release and baseline binding potential between HC users and unstaged NC women exist, but were obscured in our sample. However, data from Smith et al. (2019) suggest this is unlikely. In their study, DA release (as measured via [^18^F]fallypride paired with D-amphetamine) did not differ between women using hormonal contraception and NC women staged within the first 10 days of their menstrual cycle.

Another consideration is that FMT signal increases over the adult lifespan. Braskie et al. (2008) observed greater striatal FMT K_i_ values in older participants (mean age = 67) relative to younger participants (mean age = 23). In young adults, higher FMT K_i_ values in caudate are associated with increased working memory capacity (Cools et al. 2008). In contrast, in older adults greater striatal FMT signal may reflect potential compensation for deficits elsewhere in the DA system (e.g. PFC). In a recent study of DA synthesis and working memory capacity in cognitively normal older adults (Ciampa et al. 2021), we observed that adults with the highest FMT K_i_ values also display the greatest atrophy in posterior parietal cortex, raising the possibility of a compensatory response with aging. In the present study of younger adults, HC users were slightly older than NC participants (2 years on average), but the age range of our sample was limited (18–28 years) and results remained significant after controlling for age. Thus, it is unlikely that the group differences we observed are attributable to general effects of aging. Furthermore, our results do not support the idea that higher FMT K_i_ values reflect suboptimal DA functioning, given that HC users had higher FMT K_i_ values and greater cognitive flexibility. Higher FMT K_i_ values in young adults have consistently been associated with better cognitive flexibility (Berry et al. 2016, 2018b) as well as with working memory capacity (Cools et al. 2008).

### Consistent effects across hormonal contraceptive regimens

The women in our HC group were on different forms of hormonal contraception, including the combined oral contraceptive pill, vaginal ring, subdermal implant, injection, and hormonal IUD. Exploratory analyses suggest that the relationship between HC use and potentiated DA synthesis capacity is independent of route of administration (Supplementary Fig. 1). Similarly, in their population-level study of hormonal contraception use and mood disorders, Skovlund et al. (2016) report an increased risk with hormonal contraceptive use, regardless of method and formulation. Hormonal contraception can alter endogenous hormone concentrations to varying extents depending on the formulation and method of delivery. Oral contraceptives and the depot medroxyprogesterone injection exert powerful and sustained suppression of endogenous sex hormone production (Gaspard et al. 1983; Croxatto and Mäkäräinen 1998; Rivera et al. 1999), whereas hormonal IUDs and implants generally exert less pronounced suppression of endogenous hormone levels (Barbosa et al. 1990; Luukkainen et al. 1990; Xiao et al. 1995; Croxatto and Mäkäräinen 1998; Coelingh Bennink 2000). Therefore, the impact of HC on DA may occur in part by altering endogenous hormone levels, but the observed effects are unlikely to be solely attributable to endogenous hormone modulation, per se.

The synthetic hormones introduced by the HC regimen, not the alteration of endogenous hormones alone, may be driving changes within the DA system. In one of the few studies of synthetic hormones’ effects on striatal DA, Jori and Dolfini (1976) report increased turnover of striatal DA in intact female rats after acute and chronic oral administration of steroid contraceptive drug combinations (mestranol with either lynestrenol, norethindrone, or norethynodrel). While endogenous estrogen levels are suppressed in users of OC, the exogenous estrogen (typically ethinylestradiol) is considered to be significantly more potent than its endogenous analog (Helgason et al. 1982; Jeyakumar et al. 2011). If synthetic hormones’ potency confers an enhanced estrogenic effect (see Hampson 2023, for discussion), this could be contributing to the augmented DA synthesis we see in HC users relative to nonusers. However, this account could not fully explain the pattern of results observed here, since users of progestin-only IUDs were similarly affected (n.b. Skovlund et al. 2016).

Here, DA synthesis capacity was similarly elevated in users of OC (“the pill,” which is primarily a combination of ethinyl estradiol and progestin) and users of other forms of hormonal contraception (including implants, injection, and hormonal IUDs) that primarily contain progestin. This suggests that the progestin component, alone or in concert with endogenous or exogenous estrogen, could be influencing the observed effects. Skovlund et al. (2016) proposed that their findings of increased risk of depression across all types of HC reflected the influence of progestins. A general consensus from animal and human research is that endogenous estradiol augments DA function (reviewed in Barth et al. 2015), whereas the influence of progesterone has not been fully characterized (Hidalgo and Pletzer 2017). Still, progesterone receptor expression in embryonic DA neurons suggests a potentially powerful role of progesterone in modulating DA signaling. In a study of mouse embryonic stem cells, Díaz et al. (2007) studied the expression of steroid hormone receptors in differentiated DA neurons. They report that 92% of DA neurons expressed progesterone receptors and only 19% of these neurons co-expressed TH and ER-α. Other studies report effects of progesterone, independent of estrogens, on DA release (Dluzen and Ramirez 1990; Petitclerc et al. 1995). Future investigations delineating the influence of synthetic progestins alone and in combination with ethinyl estradiol on dopaminergic function will provide mechanistic insight into the results reported here.

### Hormonal modulation of dorsal caudate vs striatum broadly

We observed a significant difference in DA synthesis capacity between HC and NC groups across the striatum, and post hoc tests revealed the strongest effect to be in dorsal caudate (Fig. 1). Thus, hormonal contraception may broadly alter striatal DA synthesis capacity, or do so more selectively within dorsal caudate. In a case study of oral contraceptive-induced hemichorea using ^18^FDG-PET, investigators observed striatal hypermetabolism, with increased glucose metabolism in the body of the left caudate nucleus (contralateral to the dyskinesia) (Vela et al. 2004), suggesting augmented effects of OC in the caudate.

### Individual differences in DA synthesis capacity are tied to cognitive flexibility

Hormonal contraceptive users differed from NC women on switch cost but not on distractor cost in this task-switching paradigm, suggesting a specific effect on cognitive flexibility. This reduced switch cost (i.e. greater cognitive flexibility) in hormone users is consistent with our observation of greater striatal DA synthesis capacity in hormone users relative to NC women, and aligns with models of cognitive control, which posit unique roles of prefrontal and striatal DA on cognitive stability (i.e. distractor cost) and flexibility (switch cost), respectively (Cools and D’Esposito 2011). Previous studies have reported an association between task-switching performance and DA synthesis capacity, specifically in the dorsal caudate (Klanker et al. 2013; Berry et al. 2016, 2018b). Our results suggest an influence of hormonal contraceptive use on the corticostriatal circuitry underlying executive functioning. Future studies should consider whether other measures of executive functioning are influenced, and, by extension, whether dopaminergic medications used to treat disorders of executive function (e.g. ADHD) exert unique effects with or without concomitant use of hormonal contraception.

### Strengths and limitations

Together, this study provided a unique opportunity to examine differences in basal DA receptor occupancy, stimulated DA release, and DA synthesis capacity in a single cohort, based on women’s hormonal contraceptive status. However, a number of limitations should be considered. First, these data were collected as part of a parent study that was not designed with a woman’s health lens in mind. For this reason, our sample is representative of contraceptive use by the general pool of women enrolled in the parent neuroimaging study. The route of administration and formulation of the hormonal contraceptive regimen varied (e.g. patch, pill, IUD, and implant) among HC participants. Detailed information on participants’ age of initiation, duration of hormone use, and schedule would further enhance our understanding of the time course with which hormonal contraceptives impacts the DA system. Second, NC participants were not staged according to menstrual cycle phase. Thus, the robust differences we observed may reflect stable characteristics of the DA system that differ between groups as opposed to state-dependent effects sensitive to hormonal variation across the cycle. It remains a possibility that there are differences in DA signaling *within* NC women over time or between contraceptive users and NC women at specific cycle stages (as opposed to NC women generally). Finally, though we determined that a number of non-hormonal factors (e.g. working memory span, impulsivity, and smoking status) that could influence DA neurotransmission were likely not contributing to our findings, there are other factors (e.g. IQ) that future studies should also take into consideration.

This study leveraged an existing multimodal molecular PET imaging cohort, which gave us the rare opportunity to identify broad differences in DA neurotransmission between NC women and those using hormonal contraception. This analysis provides researchers with a strong rationale for conducting additional studies designed explicitly to test the breadth and depth with which hormonal contraceptives influence major neuromodulatory systems. Strikingly little has been done to investigate the impact of chronic ovarian hormone suppression and synthetic hormone regimens on brain regions that are densely populated with sex hormone receptors and modulated by sex hormones (Taylor et al. 2021; Petersen et al. 2023). This multi-tracer molecular PET imaging study, which allows a comprehensive assessment of DA receptor occupancy, DA release, and DA synthesis in a single cohort, represents a critical step toward that goal.

## Conclusions

This PET imaging study revealed differences in DA synthesis capacity between hormonal contraceptive users and naturally cycling women. Measures of DA binding potential and stimulated DA release were similar between groups. Hormonal contraception (in the form of pill, shot, implant, ring, or IUD) is used by ~400 million women worldwide (United Nations 2019), yet few studies have examined whether hormonal manipulations impact basic properties of the DA system. Findings from this study begin to address this critical gap in women’s health. Moving forward, it is important to consider hormone use as a factor in studies of DA function. More broadly, our findings motivate consideration of the clinical implications of concomitant use of commonly used DA-based medications and hormonal contraceptives.

## Funding

The National Institutes of Health AG044292 (WJ); the Daryl and Marguerite Errett Discovery Award (CMT); NARSAD Young Investigator Grant from the Brain & Behavior Research Foundation (EGJ); the Ann S. Bowers Women’s Brain Health Initiative (EGJ, MTD).

*Conflict of interest statement:* The authors report no conflicts of interest.

## Author contributions

The parent project was conceived by MTD and WJJ, with study aims conceived by CMT and EGJ; WJJ, DJF, RLW and A.S.B. and performed the experiments; data analysis was conducted by CMT and DJF; CMT and EGJ wrote the manuscript; MTD, ASB, DJF, WJJ, RLW, CMT, and EGJ edited the manuscript.

Caitlin Taylor (Conceptualization, Formal analysis, Visualization, Writing—original draft, Writing—review & editing), Daniella L. Furman (Formal analysis, Investigation, Methodology, Visualization, Writing—review & editing), Anne Berry (Investigation, Writing—review & editing), Robert L. White III (Investigation, Writing—review & editing), William Jagust (Conceptualization, Investigation, Writing—review & editing), Mark D'Esposito (Conceptualization, Writing—review & editing), and Emily Jacobs (Conceptualization, Writing—original draft, Writing—review & editing)

## Supplementary Material

Taylor_CerebralCortex_supplemental_bhad134
