# Supplementary material for: Striatal dopamine synthesis and cognitive flexibility differ between hormonal contraceptive users and nonusers
Source: Cereb Cortex. 2023 May 9;33(13):8485–95. doi: 10.1093/cercor/bhad134 (PMC10321119; doi:10.1093/cercor/bhad134)
Supplement: Taylor_CerebralCortex_supplemental_bhad134 [file taylor_cerebralcortex_supplemental_bhad134.docx]

**Striatal dopamine synthesis and cognitive flexibility differ between hormonal contraceptive users and non-users**

**Supplementary Material**

Caitlin M. Taylor^1^, Daniella J. Furman^2^, Anne S. Berry^3^, Robert L. White III^4^, William J. Jagust^5,6^, Mark D’Esposito^5,7^, Emily G. Jacobs^1,8^

^1^Department of Psychological & Brain Sciences, University of California, Santa Barbara

^2^Department of Neurology, University of California San Francisco

^3^Department of Psychology, Brandeis University

^4^Department of Neurology, Washington University School of Medicine

^5^Helen Wills Neuroscience Institute, University of California Berkeley

^6^Lawrence Berkeley National Laboratory

7Department of Psychology, University of California Berkeley

^8^Neuroscience Research Institute, University of California Santa Barbara

**
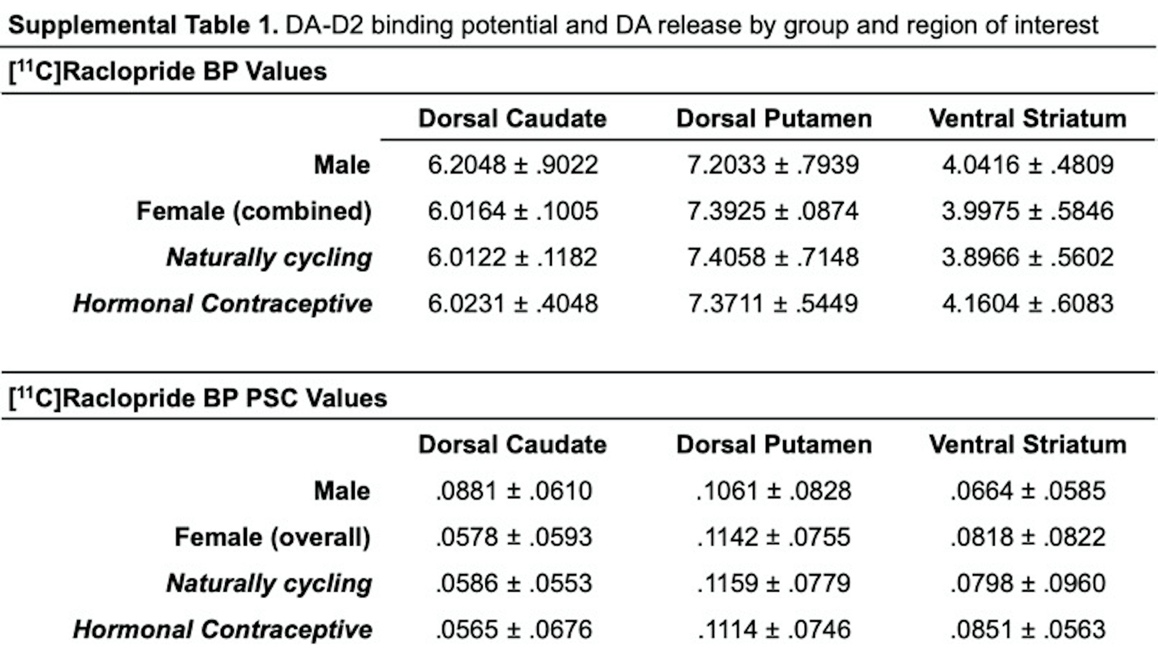
**

**Supplemental Table 1.** Striatal [^11^C]Raclopride BP values and Percent Signal Change across group and striatal region of interest.

**
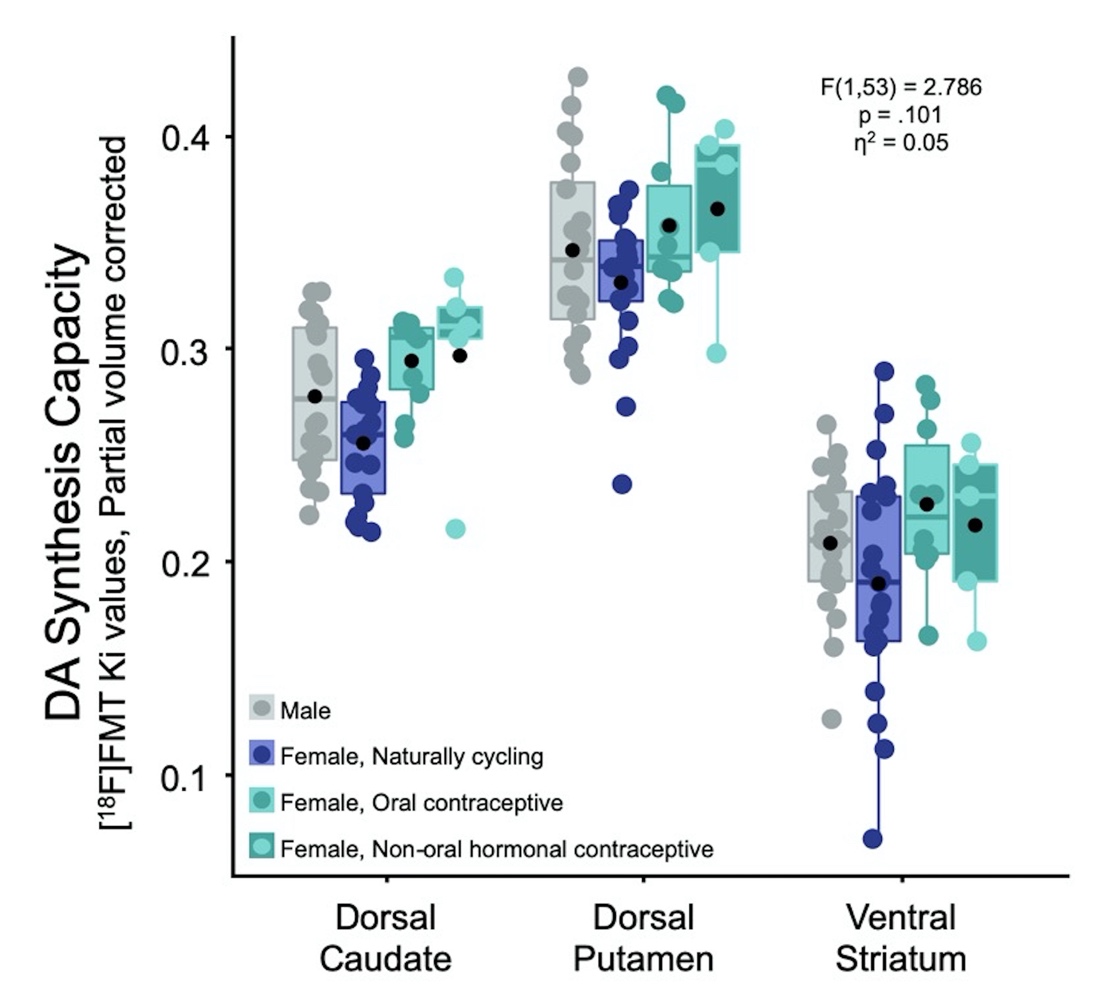
**

**Supplemental Figure 1. Effects of hormonal contraceptive use on DA synthesis capacity similar with oral and non-oral administrations.** [^18^F]FMT Ki values in males, naturally cycling females, oral contraceptive users (pill; n = 10), and hormonal contraceptive users (i.e. ring, implant, injection, IUD; n = 5), by striatal region of interest. Note that hormone users show elevated DA synthesis, despite the route of administration.

**
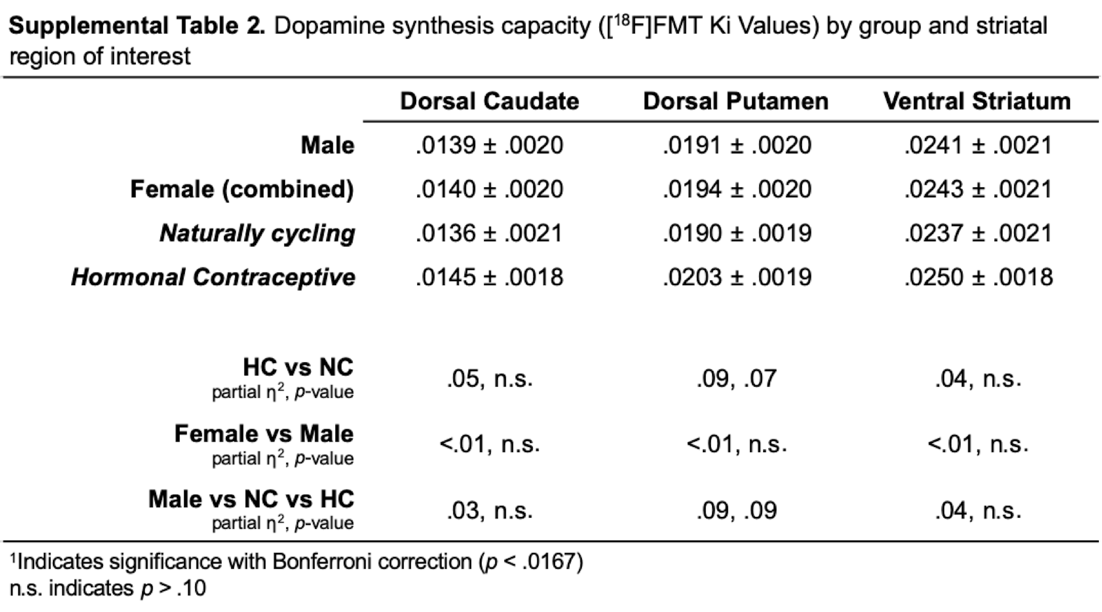
**

**Supplemental Table 2.** Non-PVC-corrected striatal [^18^F]FMT Ki values across group and striatal region of interest.
